# On the Restoration of the Alamo

**Published:** 1913-05

**Authors:** Daniel Parker

**Affiliations:** Calvert, Texas


For Texas Medical Journal.

### On the Restoration of the Alamo.

BY DANIEL PARKER, M. D., CALVERT, TEXAS.

Dedicated to Mrs. Clara Driscoll Sevier and her companions, the "Daughters of the Republic."

How shall we decorate the ground
Where Patriot heroes bled?
Whose hands shall twine a wreath around
The memory of the dead?

Shall noble woman's practiced hand
The sacred place adorn,
On which that gallant patriot band
Essayed a task forlorn!

Yes, on the spot where heroes bled
Let lovely flowers grow,

Forget-me-nots, carnations red
And roses white as snow.

Let weeping willows, walks and shade
And ornate fountains play,
And grassy plots and bowers be made,
Where little children stray.

So, if, as ancient warriors thought
Their spirits may return
To view the field where once they fought
And present aspect learn.

They may not see the blood-stained walls
On which they met their fate,
The gory ground, their comrades slain;
A spectacle of hate.

But may they see a vision fair
By lovely woman wrought,
A scene of peace and happiness,
Their precious blood has bought.

They saw in death that battlement,
And heard the cannon roar,
They need no re-establishment
To see that battle o'er.

The chapel stands, a fitting shrine,
And shall forever be
A monument made more divine
By our Thermopylae.

So not in vain the sacrifice,
While time in cycles rolls,
We'll beautify this sacred ground
And pray; God rest their souls.

